# Evaluation of effectiveness of school-based nutrition education in improving the consumption of pulses-based food among female adolescents in Northwest Ethiopia: a cluster randomized controlled trial

**DOI:** 10.1186/s41043-023-00446-7

**Published:** 2023-10-17

**Authors:** Fantahun Ayenew Mekonnen, Gashaw Andargie Biks, Telake Azale, Netsanet Worku Mengistu

**Affiliations:** 1 Department of Epidemiology and Biostatistics, Institute of Public Health, College of Medicine and Health Sciences, University of Gondar, Gondar, Ethiopia; 2 Department of Health Systems and Policy, Institute of Public Health, College of Medicine and Health Sciences, University of Gondar, Gondar, Ethiopia; 3 Department of Health Education and Behavioral Sciences, Institute of Public Health, College of Medicine and Health Sciences, University of Gondar, Gondar, Ethiopia; 4 Department of Human Nutrition, Institute of Public Health, College of Medicine and Health Sciences, University of Gondar, Gondar, Ethiopia

**Keywords:** Cluster randomized controlled trial, Adolescents, Pulses, Pulses-based food, Pulses consumption, Protein consumption

## Abstract

**Background:**

Protein undernutrition is a prevalent health problem in Ethiopia severely affecting the reproductive outcome of women. This is mainly because of inadequate consumption of protein due to the high cost of animal-origin food and the lack of knowledge about the benefits and the methods of preparation of pulses-based foods. Therefore, this trial was conducted to evaluate the effectiveness of nutrition education in improving the consumption of pulses-based foods among female adolescents.

**Methods:**

A two-arm pragmatic cluster randomized controlled trial was conducted among female adolescents in Northwest Ethiopia. Clusters were schools assigned into intervention and control groups by cluster randomization. The trial participants were female adolescents. The intervention was pulses-based nutrition education, and the comparator was the usual dietary practice of adolescent girls. The education was delivered over four weeks on a 45–60 min session per week basis. The primary outcome of the intervention was pulses-based food consumption, and the secondary outcomes were knowledge and attitude about pulses food. Data on the outcome and the confounding variables were collected at baseline and end-line of the intervention. The analysis was based on intention-to-treat analysis, and a log-binomial logistic regression model was fitted to the data to calculate relative risk with the corresponding *p* value adjusted for baseline characteristics. The intervention was considered effective when the *p* value was < 0.05.

**Results:**

A total of 269 intervention and 278 control participants from the four clusters completed the trial making response rates of 92.1% and 95.2%, respectively. The pulses-based nutrition education enabled participants in the intervention group to maintain their pulses-based food consumption state, while participants in the control group significantly reduced their consumption by about threefold [ARR; 95% CI 2.99 (1.87, 4.79)] from harvesting to non-harvesting season. The consumption of pulses-based food was higher by 16% among the intervention participants as compared to the control participants [ARD; 95% CI 0.16 (0.10, 0.21)].

**Conclusion:**

Pulses-based nutrition education is effective in improving the consumption of pulses-based food among female adolescents. Therefore, policies and strategies are required to integrate this intervention in the school nutrition program.

*Trial registration*: The trial was registered in the Pan African Clinical Trials Registry (PACTR202111813445259) on 02 November 2021.

## Introduction

Adolescent girls’ protein undernutrition continues to be one of the most important public health burdens in Low- and Middle-Income Countries (LMICs) [1–4]. Analysis of worldwide trends in body-mass index (BMI), underweight, overweight, and obesity from 1975 to 2016 showed that the mean BMI estimates for youths aged 10–19 in South Asia, South-East Asia, East Africa, West Africa, and Central Africa were < 20. According to the report, Ethiopia is among countries like Niger, Senegal, India, Bangladesh, Myanmar, and Cambodia, where the lowest BMIs in the world are observed [5]. The Ethiopian Demographic and Health Survey (EDHS) has also reported that 29% of adolescent girls in Ethiopia are thin [6]. There are also similar studies in the different parts of this country that reported thinness and stunting prevalence ranging from 13.6–29% [7–12] to 11.9–47.4% [9, 10, 12–15], respectively.

Protein-energy undernutrition in adolescent girls puts them at increased risk of morbidity and mortality, further affecting their reproductive outcomes. This is because undernourished adolescents tend to be ultimately undernourished mothers making them give birth to small babies [16]. The low birth weight baby, in turn, is more vulnerable to several risks of illness and premature death [17, 18], developmental problems of delayed physical and mental development and reduced intellectual capacity leading to poor educational achievement, school attendance and concentration, perpetuating the cycle of undernutrition across generations. In this way, undernutrition is perpetuated across generations and heavily affects the productivity of a given nation [19, 20]. However, adolescence is the second opportunity to correct the nutritional deficiencies experienced by children in their early life and break the intergenerational cycle of undernutrition [21].

Even though several factors are responsible, inadequate protein intake is believed to be the most important cause of adolescent girls’ thinness and stunting in LMICs. This is mainly due to protein source food inaccessibility and/or poor knowledge and attitude about nutrition [1, 22–25]. In this regard, animal-source protein foods like meat, fish and dairy food intake are low in Africa and are expensive for the majority of Africans, including Ethiopians [26–30]. On the contrary, however, plant-source proteins or pulses like chickpeas, lentils, peas and broad beans are known to be rich in protein and are much more affordable. They are the most commonly produced crops worldwide, and Ethiopia is among the top 10 pulses crop producing countries globally [31–33]. When combined with cereals, they can supply the amino acids necessary for growth and development. As a result, they are highly recommended to be consumed daily as an alternative to animal-origin foods [31, 34]. However, its consumption is very low and should be improved in the country to benefit from its high nutrient value [35–37]. Hence, this study was conducted to evaluate the effectiveness of pulses-based nutrition education in improving the consumption of pulses-based food among female adolescents.

## Methods

### Trial design

This study was a pragmatic stratified cluster randomized parallel controlled trial. The clusters were schools, and the participants were female adolescents. The study had two arms, intervention and control arms. A total of four schools were purposively selected and assigned to the intervention group (IG) and control group (CG) by using a stratified cluster randomization technique. Female adolescents were then recruited by systematic random sampling from the respective schools. The intervention was pulses-based nutrition education, while the comparator was the usual dietary practice of female adolescents. Participant recruitment was conducted in the 1st week of December 2021, and baseline data were collected in the 3rd week of December 2021. The nutrition education was conducted from the 4th week of December 2021 to the 3rd week of January 2022, and end-line data were collected in the 1st week of June 2022.

### Participants

The present trial enrolled a total of 584 female adolescents (292 in each group) who are 15–19 years of age, attending grades 9–12 at the four schools (clusters) located in Central Gondar Zone, Northwest Ethiopia. Only female adolescents who were willing to participate were included in the study. Those participants who were unable to communicate and had a plan to leave the study area or school before the completion of the study were excluded from the trial. Concerning clusters, only schools that are located in areas where pulses production is common have been included. On the other hand, whenever schools are considered to be close to each other, the one situated between schools was excluded and considered as a buffer zone to minimize information contamination.

### Study setting

The study was conducted in the Central Gondar zone, which is located about 726 km away from Addis Ababa, in northwest Ethiopia. The zone has 15 districts and 29 secondary schools. About 46,340 students were attending their education in these schools during the study period. The zone has two climatic conditions, highland and lowland. The highland climatic area occupies the north and some part of the east of the zone, while the lowland occupies the south, west and remote east of the zone. Accordingly, the areas are different in pulses crop production. Pulses crop production is common in the north and south parts of the zone. On the contrary, production is rare in the western part of the zone. Though not as common as in the north and the south, pulses are also produced in the East. The types of pulses crops produced are different between the North and the South due to their difference in climatic conditions. The most common pulse crops produced in the North are peas and broad beans, and chickpeas in the South. There is also a difference in the zone in food security status. The north and the eastern parts of the zone are relatively food insecure as compared to the south and the west (communications with Central Gondar Zone Education and Agriculture offices). The study was conducted from December 2021 to June 2022.

### Intervention

The intervention was a pulses-based nutrition education delivered to female adolescents from the two schools. The comparator was the usual dietary practices of female adolescents from the other two schools. The female adolescents from the intervention arm received weekly lessons for four weeks on one session per week basis, each session lasting for about 45–60 min.

The first session was about the definition of undernutrition and the short and long-term consequences of undernutrition for female adolescents. The second session was an overview of food groups that constitute a balanced diet, including their sources and nutritional functions. The third session emphasized common pulses in the study area, such as broad beans, peas, chickpeas and lentils. This session included the daily required quantity of pulse and the details of pulse processing, which included cleaning, washing, soaking, germination and boiling, and the benefit of further processing of pulses relating to removing anti-nutrients, improving its test and digestibility, and mixing them with cereals for improving the quality of proteins. All the above three sessions were theoretical sessions, the teaching methods of which were lecture and group discussion with the aid of flip charts, posters and leaflets. The fourth session was a demonstration of what was detailed in the third session. Selected pulses-based foods were demonstrated during this session. The selection of recipes considered the relative ease of accessing the pulses and other ingredients and ease in terms of time and skill to prepare them. Pulses-based foods like foods in soup and solid forms mixed with rice and vegetable were demonstrated. This is because our previous survey revealed that although the consumption was less frequent and not usually mixed with cereals, the preparation in roasted, boiled, germinated and bread forms are not new in the study area. Pulses and rice were the main components in the demonstrations, with vegetables, spices and oil added to enhance the flavor of the food. The quantities of pulses mixed were made to approximate the recommended quantity. Broad beans were used for the demonstrations conducted in the North and chickpeas in the South.

The theoretical components of the nutrition education were delivered by the investigators, and the demonstrations were conducted by a chef. The maximum number of participants per training session for the theoretical and demonstration was 50.

To enhance adherence to the nutrition education sessions, participants who were unwilling to participate after an explanation of the purpose, risks and benefits of taking part in the intervention were excluded. We also excluded participants who were not sure whether they would leave the school during the study period or not. A nutrition education manual was developed and used to enhance fidelity.

The nutrition education manual was developed following a review of Food and Agriculture Organization (FAO) materials related to general dietary knowledge, attitude and practice assessment guidelines, and guidelines specific to pulses processing and pulses-based food preparation [31, 32, 38]. Pender’s Health Promotion Model (HPM) manuals and guidelines were also used [39–41]. The manual was then presented to experts, and their feedback was received and incorporated.

### Outcome of the intervention

Pulses-based food consumption was the primary outcome of the present trial and knowledge, beliefs, perceptions and experiences about pulses food, and dietary diversity were the secondary outcomes.

Pulses-based food consumption was dichotomized as yes or no levels, and it was based on 24 h dietary recall. The pulses-based food consumption was regarded as ‘yes’ if the amount of pulse the participant consumed was a cup or a handful, to approximate 100–120 g cooked pulses consumption in the past 24 h in one or more of the following forms; roasted, boiled, germinated, in bread, or mixed with cereals and/or vegetables [31, 33].

Pertaining to participants’ knowledge, beliefs, perceptions and experiences about pulses-based food, 26 questions with an acceptable item consistency (Cronbach’s alpha = 0.72), organized based on Pender’s Health Promotion Model domains, were used [41]. The domains with the respective numbers of items were as follows: [1] commitment to preparing and consuming pulses-based food (5 items, e.g.,—I am committed to preparing and eating pulses-based food in boiled forms mixed with cereals); [2] taste-related barriers to consuming pulses-based food (7 items, e.g.,—I do not eat pulses food in boiled form since I do not like its test); [3] self-efficacy beliefs related to consuming pulses-based food (6 items, e.g.,—I can plan to preparing and eat pulses in germinated form mixed with cereals); [4] perceived benefit of consuming pulses (2 items, e.g., -Eating pulses mixed can help enhance growth and development); [5] impact of interpersonal influence on consuming pulses-based food (3 items, e.g.,—My friends encourage me to consume pulses food); and [6] knowledge/skill/accessibility-related barriers to consuming pulses-based food (3 items, e.g.,—Since I do not have the knowledge, I cannot prepare and consume pulses mixed with rice). The items were presented on a 5-point Likert-type scale. In addition to Pender’s Health Promotion Model (HPM) manual, other related guidelines were also used to develop the items [39–41]. So, when the summed score of a participant’s items for a given domain was greater than the summed score of the middle value of that domain, then that participant was categorized as having favorable behavior.

On the other hand, dietary diversity was categorized as good if the trial participant reported consuming five or more of the ten food groups: cereals/roots/tubers, pulses, nuts and seeds, dairy food, meat and poultry, egg, green leafy vegetables, other vitamin A rich fruits and vegetables, other vegetables, and other fruits. Otherwise, it was considered poor dietary diversity. For a food group to be considered consumed in the past 24 h, the serving size of any item in that food group needed to be at least one table spoon or about a fistful, which is roughly equivalent to 15 g [42].

### Sample size estimation

The sample size calculation procedure involved three steps; 1st) using individual randomized study; 2nd) considering design effect; and 3rd) considering loss to follow-up or unavailable data of 15% [43]. A 15% increase was expected by the present intervention from a 9% pulses food consumption proportion from a previous study [37]. So, the double population proportion formula was used to calculate the sample size taking P1 = 9% and P2 = 24% and also assuming significant level = 0.05 and power = 0.9. The final sample size, after considering an intra-cluster correlation coefficient (ICC) of 0.03 with common cluster size and lost to follow-up 15% was 584 participants, 292 in each arm.

### Randomization

Schools were used as units for randomization into intervention and control groups. A total of four schools were randomized using stratified cluster randomization. Stratification was made by climatic condition/types of pulses commonly produced, and thus two geographic areas were formed. Two schools were then selected from each geographic area or stratum. The schools were purposively selected in such a way that they are reasonably far apart from each other. The two schools from each stratum were finally randomized into the intervention and control groups using an envelope. Each stratum/geographic area was made to have one intervention and one control school/cluster. Individual participants were then recruited using a systematic random sampling method. Study participants from the intervention schools were intervention recipients, while study participants from the control schools continued with their usual dietary practice.

### Allocation concealment and blinding

Consent and recruitment of the school and individual study participants were completed before the schools were randomized into intervention groups. Baseline data were also collected from the study participants before the schools were randomized. The personnel facilitating the self-administered questionnaire for the end-line data collection were unaware of which participant or school was part of the intervention group and which one was not. One of the academic staff in the Department of Epidemiology and Biostatistics at the University of Gondar conducted the randomization procedure.

### Data collection tools and procedures

Baseline and end-line data on socio-demographics, dietary practice, including consumption of pulses-based food and participants’ knowledge, beliefs, perceptions and experiences related to pulses-based food were collected by self-administered questionnaire.

Data on the consumption of pulses-based food were collected using the past 24 h pulses-based food consumption history adopted from the 24 h recall dietary practice assessment approach. Thus, the questions were those of FAO’s six close-ended yes or no options and six probing questions, which were now limited to pulses-based food consumption [38]. The questions had a clear instruction for the students that they should choose the yes option if the pulse they really consumed in the past 24 h was about the size of a handful or a cup [31, 33]. The probing questions were used to list the various forms of pulses-based food consumed at a particular point in time in the past 24 h.

Concerning data relating to general dietary practice, FAO’s dietary practice assessment questions, six of which were close-ended with a ‘yes’ or ‘no’ response option, and six open-ended questions were used. The six close-ended questions were about the consumption of any food and/or drink except water that had been taken in the past 24 h at six different time points and in between (early in the morning, late in the morning, noon, afternoon, evening, and before going to bed). Each close-ended question was followed by a probing question with adequate space for respondents to write on the details of what was eaten and drunk at the point in time and between, provided that the answer for the closed-ended question was yes. These dietary practice assessment questions had a clear instruction for the participants that they should choose the ‘yes’ option if the food they consumed was roughly about a size of a table spoon or a fistful, which is assumed to be equivalent to 15 g [38, 42, 44].

### Quality assurance

A number of data quality assurance activities were undertaken at the different stages of the study. Participants were enrolled in the trial after they have been well introduced to the trial objectives and the amount of time it could take to answer the questions. The participation was based completely on the willingness of the participants and their parents. Data were collected whenever they had free classes, at break time, before the day’s class begins or immediately following class ending, based on their preference. When the filled questionnaire was returned, correction for completeness, consistency and accuracy was done on the spot before the student left the room. There was an attempt to make pre- and post-test data collection contexts (timing and place) as similar as possible. Both the baseline and end-line data were entered into epi-info version 7.1 as a separate data set and exported to Stata 16.0. The two data sets were then merged using a unique but common code for both of the data sets. The principal investigators supervised the data collection. This report was prepared using the Consolidated Standards of Reporting Trials (CONSORT) guideline extension for cluster randomized trial [45].

### Statistical analysis

Chi-square testing was used to examine baseline socio-demographic differences between intervention and control groups. It was also used to examine changes between pretest and post-test measurements of primary and secondary outcome variables among the groups. Bi-variable and multivariable Log-binomial regression models were fitted to the data to examine the crude and adjusted effects, respectively, of pulse-based nutrition education in improving the consumption of pulses-based food. Subgroup analysis was done on selected pre-specified socio-demographic variables. Relative risk with confidence interval and *p* value were calculated. The effect size was considered statistically significant when the *p* value was less than 0.05. An intention-to-treat analysis was conducted. Stata version 16.0 was used for data analysis.

## Results

### Flow diagram

**Fig. 1:**
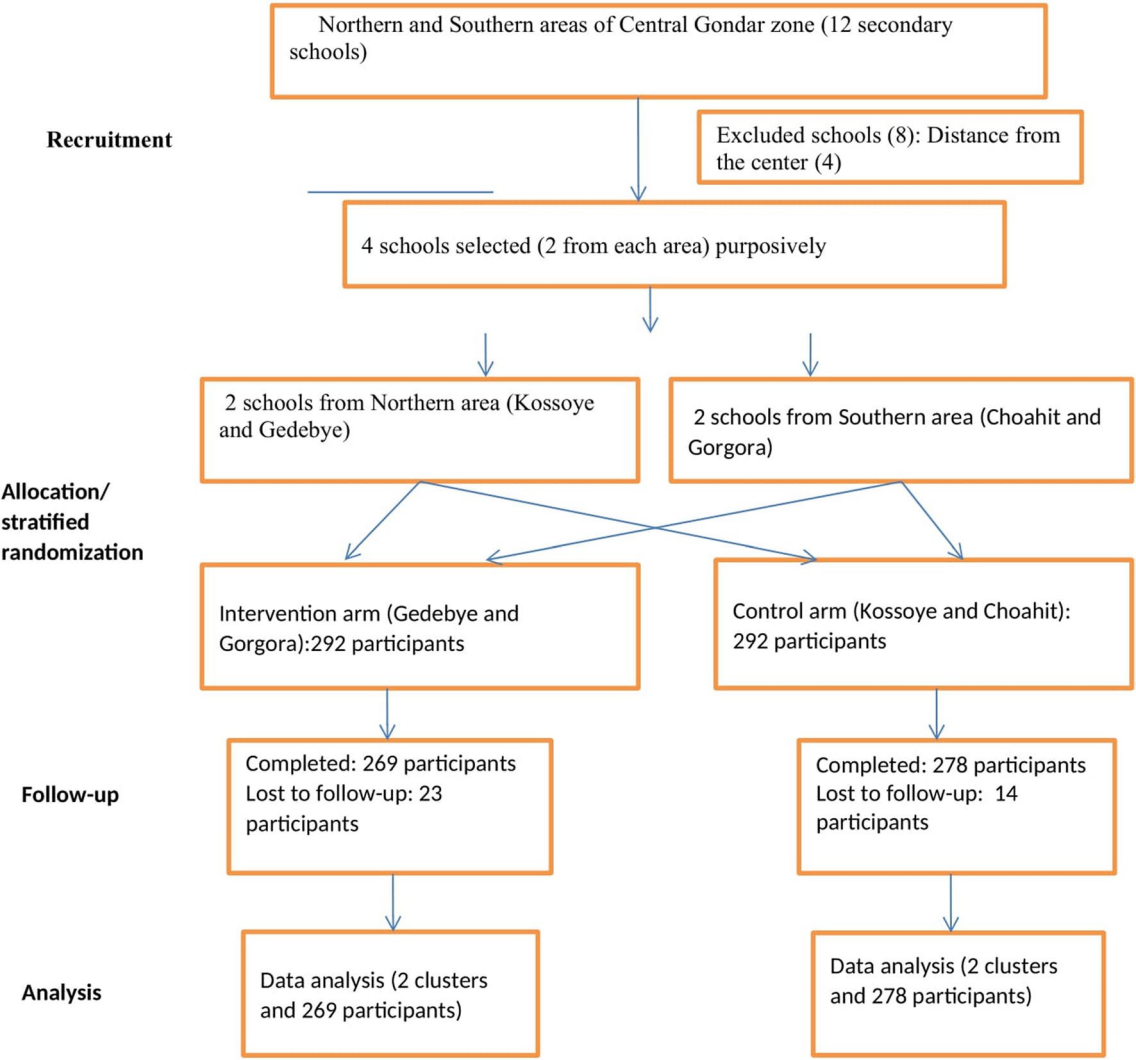
Trial flow diagram of pulses-based nutrition education compared to usual dietary practice among female adolescents, Ethiopia, 2022

A total of 584 participants, 292 from each arm, were enrolled in the trial from the four schools. A total of 269 intervention participants and 278 control participants completed the trial, resulting in 92.1% and 95.2% response rates, respectively. The type of loss to follow-up was school dropout (Fig. 1).

### Baseline socio-demographic characteristics

Of the participants who completed the trial, the majority, 82 (30.50%) were aged 18 in the intervention group, and 68 (24.46%) were aged 17 in the control group, while among the least frequent age groups, 35 (13%) were 15 in the intervention group, and 41 (14.75%) were in the 19 in the control group. About 28% percent of the intervention participants and 39.93% of the control participants were 9th grade, while 22.32% of the intervention and 14.75% of the control participants were in the 12th grade. About one-third of the intervention and below one-fifth of the control participants were permanent urban dwellers and about two-thirds of the intervention and half the control participants were temporary urban dwellers. Concerning participants’ history of school club participation, 189 (70.31%) of participants in the intervention group and 218 (78.41%) of the participants in the control group reported that they had previously participated in at least one school club activity (Table 1).

**Table 1:** Baseline socio-demographic characteristics of female adolescents in Northwest Ethiopia, 2022 (*n* = 547)

Characteristics	IG freq (%)	CG freq (%)	*X*^2^ *P* value
Age (years)			0.005
15	35 (13.00)	52 (18.70)	
16	48 (17.86)	63 (22.66)	
17	51 (18.97)	68 (24.46)	
18	82 (30.50)	54 (19.42)	
19	53 (19.72)	41 (14.75)	
Grade			0.003
9	77 (28.64)	111 (39.93)	
10	65 (24.18)	78 (28.06)	
11	67 (24.92)	48 (17.27)	
12	60 (22.32)	41 (14.75)	
Religion			0.05
Orthodox Christian	261 (97.09)	276 (99.28)	
Muslim	8 (2.98)	2 (0.72)	
Location			0.90
South of Central Gondar	136 (51.08)	142 (50.56)	
North of Central Gondar	133 (48.92)	136 (49.44)	
Permanent residence			0.000
Rural	181 (67.33)	235 (84.53)	
Urban	88 (32.74)	43 (15.47)	
Temporary residence			0.000
Rural	101 (37.57)	148 (53.24)	
Urban	168 (62.50)	130 (46.76)	
School club participation			0.03
Yes	189 (70.31)	218 (78.41)	
No	80 (29.76)	60 (21.58)	

### Dietary diversity and meal frequency

No statistically significant difference was observed among the intervention groups either at baseline or at the end-line of the intervention across eight of the ten food groups. These included cereals and roots, nuts and seeds, pulses, dairy food, meat and poultry, egg, dark green leafy vegetables, and other vitamin A rich fruits and vegetables. Only other vegetables and other fruits were significant at baseline among the intervention groups. However, other vegetable intake turned out to be insignificant at the end-line, while the other fruits continued to be significant at the end-line of the intervention between the intervention groups. Similarly, the dietary diversity score was significantly different among the intervention groups at the baseline and insignificant at the end-line of the intervention. Regarding the meal frequency, no significant difference was observed between the intervention and control groups either at baseline or end-line of the intervention (Table 2).

**Table 2:** Dietary diversity among female adolescents in Northwest Ethiopia, 2022

Food groups	Baseline			End-line		
	IG freq (%)	CG freq (%)	*X*^2^ *P* value	IG freq (%)	CG freq (%)	*X*^2^ *P* value
Cereals and roots	269 (100.00)	276 (99.28)	0.163	267 (99.26)	278	0.15
Pulses	238 (88.48)	245 (88.13)	0.9	259 (96.28)	263 (94.60)	0.35
Nuts and seeds	0 (0.00)	0 (0.00)	–	0 (0.00)	0 (0.00)	–
Diary food	25 (9.29)	40 (14.39)	0.08	11 (4.10)	11 (3.96)	0.9
Meat, poultry and fish	107 (39.78)	104 (37.41)	0.57	43 (16.04)	52 (18.71)	0.41
Egg	14 (5.22)	18 (6.47)	0.534	4 (1.53)	6 (2.21)	0.56
Dark green leafy vegetables	4 (1.50)	2 (0.72)	0.391	1 (0.38)	1 (0.36)	0.98
Other vitamin A rich fruits and vegetables	2 (0.74)	1 (0.36)	0.550	1 (0.40)	0 (0.00)	0.3
Other vegetables	4 (1.5)	0 (0.00)	0.044	2 (0.75)	1 (0.36)	0.54
Other fruits	13 (4.8)	37 (13.36)	0.001	13 (4.8)	37 (13.36)	0.001
Dietary diversity score	24 (8.92)	42 (15.11)	0.03	8 (3.00)	9 (3.24)	0.86
Meal frequency (≥ 3)	249 (92.57)	261 (93.88)	0.539	245 (91.08)	254 (91.36)	0.91

### Knowledge, beliefs, perceptions and experiences about pulses food

All six domains were not significantly different among the intervention groups at the baseline of the intervention. Similarly, only one of the six domains was found to have a statistically significant difference between the groups at the end-line of the intervention. That is, interpersonal influences on the consumption of pulse food changed from a statistically insignificant difference (*p* value = 0.49) at baseline to a statistically significant (*p* value = 0.02) at the end-line between IG and CG (Table 3).

**Table 3:** Knowledge, beliefs, perceptions and experiences about pulses food among female adolescents in Northwest Ethiopia, 2022

Variables	Baseline			End-line		
	IG freq (%)	CG freq (%)	*X*^2^ *P* value	IG freq (%)	CG Freq (%)	*X*^2^ *P* value
Commitment to prepare and consume pulses	153 (56.88)	156 (56.12)	0.86	208 (77.32)	201 (72.30)	0.18
Pulses taste-related barrier to consume pulses	44 (16.36)	59 (21.22)	0.15	45 (16.73)	57 (20.50)	0.26
Self-efficacy beliefs to consume pulses	197 (73.23)	185 (66.55)	0.09	218 (81.04)	231 (83.09)	0.53
Perceived benefit of consuming pulses	190 (70.63)	192 (69.06)	0.69	239 (88.85)	240 (89.22)	0.37
Interpersonal influence to consume pulses	93 (34.57)	104 (37.41)	0.49	101 (37.55)	79 (28.42)	0.02
Knowledge/skill/ and accessibility barriers to consume pulses	70 (26.02)	58 (20.86)	0.15	70 (26.02)	59 (21.22)	0.15

### Pulses-based food consumption

There was no significant difference among the intervention groups in terms of the consumption of five of the six pulses-based food forms, which included roasted, boiled, germinated, soup, bread, mixed with cereals and/or vegetables at the baseline of the intervention in the past 24 h. The pulses-based food that showed a significant difference at baseline among the intervention groups was bread form (*P* value = 0.008). Conversely, a statistically significant difference was observed among the intervention groups at the end-line of the intervention in four of the five pulses-based food forms. The changes were from a chi-square *p* value of 0.14 to 0.001 for boiled, 0.57 to 0.01 for germinated, 0.008 to 0.0001 for bread form, and 0.68 to 0.007 for pulses mixed with vegetables, from the baseline to the end-line of the intervention. Concerning overall pulses-based food consumption, there was a statistically significant difference among the intervention groups both at the baseline (0.004) and end-line (0.000) of the intervention (Table 4).

**Table 4:** Pulses-based food consumption among female adolescents in Northwest Ethiopia, 2022

Pulses-based food	Baseline			End-line		
	IG freq (%)	CG freq (%)	*X*^2^ *P* value	IG freq (%)	CG freq (%)	*X*^2^ *P* value
Boiled form	21 (7.81)	13 (4.74)	0.14	27 (10.04)	8 (2.89)	0.001
Germinated form	11 (4.09)	14 (5.11)	0.57	16 (5.95)	5 (1.81)	0.012
Bread form	32 (11.90)	15 (5.49)	0.008	35 (13.01)	6 (2.17)	0.000
Soup form	12 (4.48)	5 (1.83)	0.078	5 (1.86)	3 (1.09)	0.454
Mixed with vegetable	16 (5.95)	8 (2.88)	0.08	19 (7.06)	2 (0.720	0.000
Overall pulses consumption	68 (25.27)	43 (15.46)	0.004	69 (25.65)	20 (7.20)	0.000

### The effect of nutrition education in improving the consumption of pulses-based food

After adjusting for baseline characteristics, such as baseline pulses-based food consumption, school location, permanent residence and temporary residence, the pulses-based nutrition education was effective in improving the consumption of pulses-based food among female adolescents. The consumption of pulses-based food was found to be sustained among the intervention group, while it was reduced by about threefold in the control group [ARR; 95% CI 2.99 (1.87, 4.79)] (Table 5).

**Table 5:** The effect of pulses-based nutrition education in improving the consumption of pulses-based food, 2022 (*n* = 547)

Characteristics	Pulses consumption		CRR (95% CI)	ARR (95% CI)	*P* value
	Yes	No			
Baseline pulses consumption					
Yes	44	67	3.84 (2.53, 5.82)	2.98 (2.09, 4.26)	< 0.001
No	45	391	1		
Intervention					
Nutrition education	69	200	3.57 (2.17, 5.87)	2.99 (1.87, 4.79)	< 0.001
Control	20	258	1		

### Subgroup analysis

This subgroup analysis considered two predetermined variables-age and residence. The intervention seemed more effective among older adolescent girls. Concerning residence, however, nutrition education seemed to have worked relatively better among urban residents (Table 6).

**Table 6:** Subgroup analysis of the effect of nutrition education among female adolescents, Northwest Ethiopia, 2022

Variables	Pulses consumption		RR; 95% CI
	Yes	No	
Age = 15–17			
Nutrition education	32	102	2.73 (1.56, 4.77)
Control	16	167	1
Age = 18–19 ages			
Nutrition education	37	98	6.51 (2.40, 17.65)
Control	4	91	1
Residence = rural			
Nutrition education	43	138	3.28 (1.94, 5.56)
Control	17	218	1
Residence = urban			
Nutrition education	26	62	4.23 (1.35, 13.21)
Control	3	40	1

## Discussion

Thinness and stunting among female adolescents remain to be among the critical public health burdens in Low- and Middle-Income Countries (LMICs), including Ethiopia [1–4]. Inadequate protein intake is believed to be the causes, mainly due to inaccessibility to animal-origin foods and poor knowledge and attitude about nutrition [1, 8, 24, 27, 29, 46]. In this regard, pulses are good sources of protein and recommended to be consumed as an alternative protein source food [31–33]. However, studies, including our baseline survey, showed low consumption of pulses-based food [37, 47]. The aim of the present trial was, therefore, to examine the effectiveness of pulses-based nutrition education in improving the consumption of pulses-based food among female adolescents in Northwest Ethiopia. The trial resulted in a significant difference in the consumption of pulses-based food between the intervention and control groups. In the current trial, the consumption of pulses-based food remained the same among the intervention group, while it was decreased by about threefold in the control group [ARR; 95% CI 2.99 (1.87, 4.79)] from baseline to end-line of the intervention. This is due to the fact that the baseline outcome was measured in one of the months of the harvesting season (December), while the end-line outcome was measured in one of the months of the non-harvesting season (June).

In Ethiopia, June is known to be one of the months in the non-harvesting season in which most Ethiopians residing in the northern part of the country enter into a state of food stress [48, 49]. This can imply that had we assessed the end-line consumption in the harvesting season, we would have found a significantly higher pulses food consumption proportion at the end-line compared to the consumption at baseline in the intervention group. In this sense, the current finding could be understood that the intervention improved pulses food consumption among the intervention group compared to the control group since it saved the intervention participants from the real consumption status, which would have been the reverse, had they not received the intervention.

The current finding is in line with the finding of a study conducted among female adolescents in the South Nations Nationalities and People’s Regional State (SNNPR), Ethiopia, that reported a significant improvement in the consumption of pulses-based food among participants in the intervention group as compared to their control counterparts [50]. It is also congruent with other pulses-based nutrition education studies conducted in SNNPR, Ethiopia, reporting that pulses-based nutrition education improved the use of pulses in complementary feeding of children [51–53].

In the present study, significant improvement was observed among intervention group participants at the end-line of the intervention in the consumption of pulses in boiled, germinated, and mixed with rice and/or vegetable forms as compared to the control participants, while there was no difference in the consumption of pulses in soup form among the intervention groups, which is similar with findings of a study in SNNPR, Ethiopia [52]. In the current trial, commitment to preparing and consuming pulses-based foods significantly increased among intervention group compared to the control group, which is similar with trials conducted in Iran [54]. Our subgroup analysis, on the other hand, showed that the intervention worked better among older and current urban resident participants. There are studies that compliment this study that socio-demographic factors are important predictors of dietary behavior [28, 55]. This seems obvious that older adolescents and urban residents can better understand the intervention messages, and have the freedom, authority and confidence to prepare and consume quality food due to mainly their relative access to health information [56, 57].

The reason why the present and those previous intervention studies were effective could be due to the fact that all of the nutrition education intervention studies targeted the locally available and relatively affordable food, focusing on the main barriers of pulses-based food consumption relating to pulses food knowledge and attitude mainly about its preparations and benefits. There are plenty of studies that reported protein source foods of animal origin like meat, egg and dairy are very expensive and inaccessible by most populations in Africa, including Ethiopia [27, 29]. The improvements in pulses-based food consumption among the intervention groups in the present study might also in part be attributed to the use of the health promotion model to guide the intervention although only one of the domains of the health promotion model showed a significant improvement among the intervention group as compared to the control group from the baseline to the end-line of the intervention. The use of theories or health promotion models in an intervention study targeting behavior change is increasingly being promoted due to their role in enhancing the effectiveness of an intervention [58].

The trial has the following strengths; cluster stratification was applied to minimize cluster-level variability between intervention and control groups, and the effect size of the intervention was adjusted by the consumption of pulses-based food at baseline, school location and participant residence. In addition, this one month nutrition education intervention is more feasible to scale up than those extended interventions. Moreover, the study collected the end-line data in the 4th month from the date of the last nutrition education session, which might have minimized the differential reporting bias due to recall of what has been advised during the nutrition education.

## Conclusion

The present trial revealed that the pulses-based nutrition education intervention significantly improved pulses-based food consumption among female adolescents. So, we recommend policymakers and programmers to integrate this intervention in the school nutrition program.

## Data Availability

Not applicable.

## Abbreviations

BMI: Body mass index
CONSORT: Consolidated Standards of Reporting Trials
EDHS: Ethiopian Demographic and Health Survey
FAO: Food and Agriculture Organization
HPM: Health promotion model
CRT: Cluster randomized trial
IG: Intervention group
CG: Control group
LMICs: Low- and middle-income countries
PHPM: Pender’s health promotion model
